# Subtype-Specific Metabolic Patterns in Staging Breast Cancer: Insights from Conventional and Parametric PET Radiomics

**DOI:** 10.3390/cancers18132107

**Published:** 2026-06-29

**Authors:** Sara Calzolai Lettieri, Jelena Jandric, Lidija Antunovic, Marcello Rodari, Carmen Criscitiello, Laura Evangelista, Alessia Artesani

**Affiliations:** 1 Department of Biomedical Sciences, Humanitas University, Via Rita Levi Montalcini 4, Pieve Emanuele, 20072 Milan, Italyalessia.artesani@hunimed.eu (A.A.); 2 Nuclear Medicine Unit, IRCCS Humanitas Research Hospital, Via Manzoni 56, Rozzano, 20089 Milan, Italy; jelena.jandric@humanitas.it (J.J.);; 3 Breast Medical Oncology Unit, IRCCS Humanitas Research Hospital, Via Manzoni 56, Rozzano, 20089 Milan, Italy

**Keywords:** breast cancer, dynamic PET/CT, Patlak imaging, triple-negative breast cancer, Luminal breast cancer, parametric imaging

## Abstract

**Background:** Breast cancer (BC) is a biologically heterogeneous disease, with molecular subtypes differing fundamentally in terms of aggressiveness, metabolic behavior, and therapeutic response. Conventional ^18^F-FDG PET provides semi-quantitative assessment of glucose-analogue uptake using the standardized uptake value (SUV), whereas dynamic PET with Patlak analysis can estimate kinetic parameters such as the net influx rate constant (*K_i_*) and the volume of distribution (*V_d_*). However, the interpretation of these parameters in breast tissue remains insufficiently defined, and data across BC subtypes are limited. This proof-of-concept study explored the feasibility of deriving preliminary reference ranges for non-pathological breast tissue and describing subtype-specific patterns of kinetic parameters. **Methods**: Six breast cancer patients affected by Luminal (*n* = 4) or triple negative BC (TNBC, *n* = 3) and six control patients underwent dynamic ^18^F-FDG PET/CT. Patlak kinetic analysis generated parametric *K_i_* and *V_d_* maps. Lesion-level first-order radiomics features were extracted for each breast lesion. Non-lesional breast volumes (contralateral breast and breast excluding disease) were characterized to derive exploratory physiological reference ranges. **Results**: In control whole-breast tissue, median values were SUV 0.28 g/mL, *K_i_* 0.056 mL/min/100 mL, and *V_d_* ~9%. Non-lesional breast tissue in BC remained statistically concordant with controls. Lesion analysis suggested a possible metabolic gradient across subtypes: Luminal B with low proliferation activity exhibited lowest *K_i_* (0.34 mL/min/100 mL), and highest *V_d_* (~39%); Luminal A showed intermediate *K_i_* (1.93 mL/min/100 mL) and *V_d_* (~27%) values; TNBC showed higher *K_i_* (3.02 mL/min/100 mL) with a greater proportion of zero-valued *V_d_* voxels. **Conclusions**: This exploratory study suggests that parametric maps may be feasible for describing physiological breast tissue kinetic ranges and for exploring subtype-associated metabolic differences in BC. The observed *K_i_* and *V_d_* patterns may reflect differences in glucose phosphorylation and tracer distribution across tumor phenotypes, but the biological meaning of Patlak-derived parameters requires further methodological and histopathological validation. Larger prospective studies are needed before these parameters can be considered clinically applicable imaging biomarkers.

## Simple Summary

Breast cancer can behave differently depending on its biological subtype, but current imaging methods mainly measure how much glucose-analogue tracer a tumor takes up at one time point. Dynamic positron emission tomography may provide additional information on how the tracer enters and is retained in tissue over time. In this exploratory study, we used dynamic imaging to describe preliminary normal breast tissue values and to examine whether breast cancer subtypes show different metabolic patterns. The results suggest that non-cancerous breast tissue has broadly consistent kinetic values, while tumor lesions may show subtype-related differences, particularly in tracer uptake and retention. Because the study included only a small number of patients, these findings should be considered preliminary. They may help guide future research on dynamic imaging biomarkers in breast cancer and support larger studies linking imaging results with tumor biology and treatment response.

## 1. Introduction

In breast cancer (BC), conventional Fluorodeoxyglucose (^18^F-FDG) positron-emission tomography/computed tomography (PET/CT) is primarily indicated for staging and restaging in patients with locally advanced disease or suspected metastatic spread [1,2]. Its value lies in its whole-body assessment of metabolically active lesions, including extra-axillary nodal involvement and distant metastases [3,4]. Conversely, other imaging modalities such as mammography, ultrasound, and MRI remain frequent in disease prevention, local staging and treatment response assessment [5].

Despite this well-circumscribed clinical role, a substantial body of research has demonstrated the potential relevance of semi-quantitative PET-derived metrics, particularly in the context of treatment response assessment and prognosis prediction [6,7]. Standardized uptake value (SUV) and its changes during therapy (ΔSUV) have been associated with pathological complete response and survival outcomes, especially in aggressive subtypes such as triple-negative breast cancer (TNBC) and HER2-positive disease [8,9,10]. Additionally, recent studies have shown that baseline SUV metrics can help stratify risk and guide treatment intensification in early-stage disease [11,12]. Nonetheless, these metrics are not formally integrated into treatment planning or response-adapted strategies, largely due to variability in methodology, lack of standardization, and inherent limitations in SUV’s biological specificity [13].

A key limitation of SUV is its semi-quantitative nature. SUV is derived from detector count rates normalized to injected activity and patient-specific factors such as body weight, making it susceptible to technical variables [14,15]. More fundamentally, SUV lacks specificity as a biomarker of tumor biology because it integrates only the late-phase equilibrium uptake without providing information about the underlying physiological processes governing tracer kinetics. Specifically, it cannot distinguish between rapid irreversible glucose phosphorylation and reversible tracer distribution/perfusion effects [16]. This mechanistic opacity limits SUV’s utility for understanding the pathophysiological basis of malignancy.

In this context, dynamic FDG PET acquisition offers a mechanistically superior alternative by enabling the estimation of biologically meaningful kinetic parameters through mathematical modelling of tracer behavior over time [17,18]. Among kinetic analysis approaches, Patlak graphical analysis has been extensively validated and is widely used in clinical research to derive parametric images of the net influx rate constant (*K_i_*), reflecting irreversible trapping of phosphorylated FDG, and the distribution volume (*V_d_*), representing the reversible fraction of non-metabolized tracer accessible within the tissue region [19]. These parameters aim to provide more direct physiological characterization of tissue glucose metabolism compared with static SUV measurements, with the potential to capture biological features [20,21].

In BC oncology, dynamic FDG PET may potentially support assessments of residual metabolically active tumors after systemic therapy, early predictions of complete pathological response during neoadjuvant treatment, and risk stratification based on kinetic rather than static uptake parameters [22]. Nonetheless, translating dynamic PET kinetics into clinical application requires robust interpretation frameworks and physiological contextualization of these parameters. Specifically, reference ranges for normal breast tissue are not well established, and systematic data on *K_i_* and *V_d_* distributions across different BC subtypes remain limited. Without such contextual information, the interpretation of dynamic PET parameters remains uncertain, and their integration into clinical workflows is still premature.

In order to move beyond the abovementioned limitations, this proof-of-concept study aimed to explore the feasibility of: (1) deriving physiological reference distributions for SUV, *K_i_*, and *V_d_* in normal breast tissue and non-lesional tissue in patients with BC; and (2) describing lesion-level *K_i_* and *V_d_* distributions across distinct BC subtypes. These analyses are intended to generate hypotheses for future studies and provide insights into clinically applicable quantitative biomarkers.

## 2. Materials and Methods

### 2.1. Breast Cancer and Control Cohort

In this study, we selected six patients with BC from a larger cohort investigation conducted at our institution focused on dynamic PET imaging in oncology (Approval Ethics Committee ID: NUNC-001-2024, Prot. No. 351/24). Patients with histologically confirmed BC were recruited from individuals referred for ^18^F-FDG PET/CT examinations for oncological staging at the Nuclear Medicine Department of IRCCS Humanitas Research Hospital. Eligible participants met the following inclusion criteria: (1) suspected or confirmed BC with histopathologic diagnosis via biopsy; (2) completed dynamic ^18^F-FDG PET/CT imaging; (3) age ≥ 18 years; (4) written informed consent. Exclusion criteria were: (1) age < 18 years; (2) pregnancy or lactation; (3) inability to tolerate PET/CT imaging; (4) prior neoadjuvant therapy or systemic treatment affecting lesion metabolic phenotype.

From the larger cohort, six female patients were enrolled to establish physiologic reference distributions for normal breast tissue, and they were considered in this study as the control cohort. These individuals had undergone ^18^F-FDG PET/CT imaging for non-breast oncological indications at the same institution and were selected based on: (1) absence of current or prior breast malignancy; (2) normal mammographic and imaging assessment of breast tissue with no focal lesions; (3) no history of neoadjuvant or systemic therapy; (4) written informed consent for inclusion in research.

### 2.2. PET/CT Acquisition

**Figure 1:**
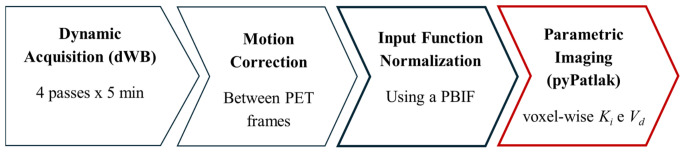
Workflow for short-dynamic acquisition and parametric image generation.

Parametric images were generated using an acquisition and reconstruction protocol validated in a previous study [23] and based on similar research [24,25,26,27]. As illustrated in Figure 1, the clinical workflow consisted of the following steps: (i) acquisition of short-dynamic ^18^F-FDG PET/CT; (ii) motion correction of PET frames; (iii) derivation of an image-derived aorta input function (AIF) from each patient and scaling of a center-specific population-based input function (PBIF); and (iv) generation of Patlak-derived parametric maps using a stand-alone processing tool.

In detail, the short-dynamic whole-body PET/CT acquisition was performed using a short-axial-field-of-view scanner (Biograph Vision 600, Siemens Healthineers, Erlangen, Germany) starting from 50 min post-injection. The protocol consisted of four consecutive whole-body passes of 5 min each, for a total dynamic acquisition time of 20 min. From each PET acquisition, both dynamic PET images and standard SUV image were reconstructed. Dynamic images were reconstructed as a series of single PET frames, whereas standard SUV images were reconstructed from the conventional static acquisition acquired 60 min after the tracer injection.

Before the Patlak graphical analysis, the dynamic PET frames were corrected for motion, in agreement with previously published work [28]. Motion correction was performed using the open-source Phyton-based FALCON software (version V2) [29,30]. In this study, deformable registration was performed with 50 × 50 × 50 iterations at each level of the multi-scale registration. The registration started from the first frame and used the last frame as a reference, to better guarantee correct co-registration between parametric results and SUV images [31].

To generate parametric maps, a center-specific PBIF was scaled to each patient using an image-derived AIF obtained from descending aorta. The AIF mask was generated by manually drawing a circular region of interest (approximately 15 mm in diameter) in the descending aorta, in agreement with previously published studies [25,32,33]. For each patient, the selected vascular region was visually inspected to exclude significant motion artifacts affecting the AIF extraction.

After motion correction of the PET frames and patient-specific normalization of the input function, Patlak parametric images were generated using an indirect reconstruction workflow, as motion correction required data export and frame-wise processing. In this study, we used an open-source Python pipeline (pyPatlak) to compute voxel-wise *K_i_* and *V_d_* maps from motion-corrected dynamic PET frames [34]. The code was previously validated against commercial direct Patlak analysis and the source code is available on GitHub (version 1.0) [35].

### 2.3. Lesion and Breast Region Delineation

Tumoral lesions were delineated on static SUV images by a board-certified nuclear medicine physician using a 40% SUVmax threshold in the Siemens Healthineers syngo.via workstation [36,37]. The resulting volume of interest (VOI) was visually reviewed and then transferred to the co-registered *K_i_* and *V_d_* maps. The presence and the molecular subtype of each breast tumor lesion were confirmed via biopsy.

In addition to tumor lesions, two non-lesional breast VOIs were analyzed as representative regions of non-pathological breast tissue. First, whole-breast masks were generated from the co-registered CT using the open-source TotalSegmentator tool [38]. From these masks, we isolated the contralateral breast, when free of tumors, and the tumor-bearing breast after exclusion of the lesion mask. In the case of bilateral disease, each tumor-bearing breast was analyzed independently at the breast level; contralateral analysis was omitted when the opposite breast also contained tumors. This strategy allowed for the inclusion of a bilateral patient (pt06) with different molecular subtypes in the two breasts.

### 2.4. Statistical Analysis

This study was designed as a proof-of-concept physiological characterization study. Accordingly, the statistical analysis prioritized descriptive range estimation, and it established preliminary reference ranges for dynamic PET parameters across distinct BC subtypes while acknowledging the exploratory nature and limited sample size of the cohort.

First-order radiomic features—including volume, mean, median, minimum, maximum, standard deviation, variance, kurtosis and skewness—were extracted from each VOI. Continuous variables were summarized using the median and interquartile range (IQR) [Q1–Q3] as primary descriptive statistics. For the characterization of physiological breast tissue metabolic values, voxel-level distributions were used to visualize the shape and range of SUV, *K_i_*, and *V_d_* values. Whole-breast voxel data were initially evaluated separately for each control subject. Inter-subject distributional concordance was assessed using the Jensen–Shannon distance and overlap coefficients (Sørensen index). Median Jensen–Shannon distance values ≤ 0.30 and median overlap coefficients ≥ 0.70 were interpreted as evidence of sufficient inter-subject concordance.

Lesion-level parameters were summarized separately for each molecular subtype. For the primary group comparison (Luminal [A + B combined] vs. TNBC), group differences were described using medians and interquartile ranges and quantified using an exact permutation test on the median difference, Hodges–Lehmann location shift with bootstrap 95% confidence interval, and Cliff’s delta effect size, also expressed as probability of superiority (P[TNBC > Luminal]). In addition, ordered subtype differences across Luminal B, Luminal A, and TNBC were explored using an exact Jonckheere–Terpstra-type trend test.

All statistical analyses were performed using Python 3.5 with scipy.stats (v1.10+) and numpy (v1.24+) libraries.

## 3. Results

### 3.1. Study Population

The BC cohort comprised six patients (one with bilateral disease, contributing seven lesions total) with a median age of 62 years (range: 50–77 years). Demographic and clinical characteristics are summarized in Table 1. The cohort was postmenopausal in five out of six cases (83%), with only one premenopausal patient. Tumors were classified into three molecular subtypes according to standard immunohistochemical criteria (ER, PgR, HER2 status) and Ki-67 proliferation index, using St.-Gallen-like surrogate definitions [39,40,41]:

- Luminal-A-like (*n* = 2): Characterized by ER-positive/HER2-negative disease with high PgR expression (≥20%) and low proliferation (Ki-67 < 20%).
- Luminal-B-like (*n* = 2): Characterized by ER-positive/HER2-negative disease with either lower PgR expression (<20%) and/or higher proliferation (Ki-67 ≥ 20%).
- Triple-Negative Breast Cancer (TNBC) (*n* = 3): Characterized by absence of ER and PgR expression and lack of HER2 overexpression/amplification, typically associated with high proliferation.

According to these surrogate criteria, a HER2-negative luminal tumor was classified as Luminal-B-like when PgR was low (<20%) and/or Ki-67 was high (≥20%); accordingly, there were two Luminal-B-like lesions in this cohort qualified on the basis of low PgR expression despite a low Ki-67 index.

In the BC cohort, HER2/neu expression was predominantly negative (score 0–1+) or equivocal (2+); all equivocal cases showed fluorescence in situ hybridization (FISH)-negative results, confirming the absence of HER2 gene amplification. Histological grading was predominantly intermediate to high (Nottingham grades 2–3) across the cohort.

The control cohort had a median age of 57 years (range: 40–68 years), which was comparable to the BC patient cohort (median 62 years; *p* = 0.512, Mann–Whitney U test). Controls were recruited for a variety of non-breast oncological indications, including pulmonary neoplasia (*n* = 1), mediastinal lymphoma (*n* = 1), colorectal adenocarcinoma (*n* = 2), cervical squamous-cell carcinoma (*n* = 1), and Hodgkin lymphoma (*n* = 1). All controls had no clinical or imaging evidence of metastatic breast involvement. Menopausal status was not systematically documented in the control cohort; however, median age suggests a mixed pre- and postmenopausal population, consistent with the BC cohort demographics.

### 3.2. Metabolic Characterization of Normal Breast Tissue

**Figure 2:**
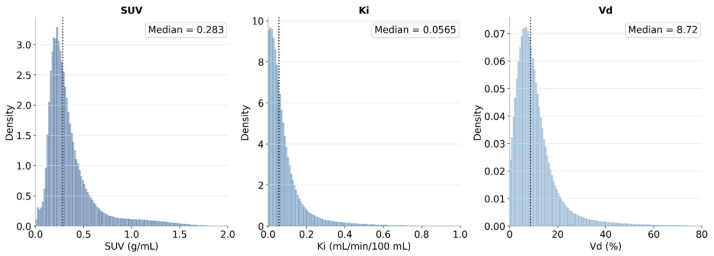
Pooled control whole-breast voxel distribution, in the three metabolic maps: SUV (g/mL), *K_i_* (mL/min/100 mL) and *V_d_* (%). Dashed vertical line is the median of the distribution.

Voxel-level distributions of all three parametric maps in control breast tissue exhibited substantial departures from normality, characterized by rightward skewness and heavy tails indicative of leptokurtic distributions (Figure 2). At the population level (pooled across *n* = 6 control subjects), standardized FDG uptake value (SUV) demonstrated a skewness coefficient of 2.43 with kurtosis of 8.04. The tracer influx parameter (*K_i_*) exhibited greater non-Gaussianity, with skewness of 2.90 and kurtosis of 11.37. The distribution volume parameter (*V_d_*) similarly showed rightward skewness of 2.78 and pronounced kurtosis of 12.23.

At the individual subject level, the degree of non-Gaussianity exhibited relative consistency across controls. Median [interquartile range] skewness values were 1.77 [1.54–2.32] for SUV, 2.20 [1.83–2.23] for *K_i_*, and 1.12 [1.03–1.24] for *V_d_*. Correspondingly, median kurtosis values were 5.16 [4.61–11.94], 7.55 [5.86–7.97], and 1.85 [1.62–2.12], respectively.

Despite departures from normal distribution, inter-individual distributions demonstrated sufficient concordance to support the derivation of exploratory population-level reference values. Inter-subject distribution similarity metrics revealed the highest concordance for *K_i_*, with a median Jensen–Shannon distance of 0.19 (range of pairwise distances: 0.16–0.23) and median overlap coefficient of 0.83. SUV; *V_d_* exhibited more substantial between-subject variability, with median overlap coefficients of 0.69 and 0.75, respectively. These findings indicate that moderate between-subject heterogeneity, particularly evident in *V_d_*, does not preclude the identification of central tendencies suitable for reference purposes.

Population-level reference distributions for control whole-breast voxels yielded the following estimates (median [IQR]): SUV = 0.28 [0.20–0.42] g/mL; *K_i_* = 0.056 [0.026–0.108] mL/min/100 mL; and *V_d_* = 8.72 [5.20–13.62] % (Table 2).

### 3.3. Parametric Distribution of Contralateral and Non-Lesional Tumoral Breast Tissue

**Figure 3:**
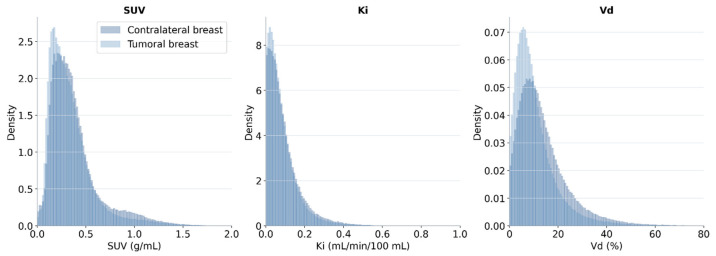
Pooled contralateral and tumoral non-lesional breast voxel distribution, in the three metabolic maps: SUV (g/mL), *K_i_* (mL/min/100 mL) and *V_d_* (%).

To contextualize normal tissue metabolic behaviors in our BC cohort, we next examined whether voxel-level distributions in contralateral breast and non-lesional tissue exhibited metabolic patterns consistent with the control reference population. Like normal breast tissue, both regions demonstrated non-Gaussian, right-skewed distributions with positive kurtosis across all three parametric maps (Figure 3); however, the magnitude of distributional deviation differed substantially between regions and parameters.

In contralateral breast tissue (*n* = 6 patients), pooled (across-patient) distributional parameters yielded skewness values of 1.40 (SUV), 1.76 (*K_i_*), and 1.14 (*V_d_*), with corresponding kurtosis values of 3.31, 4.71, and 1.85, respectively. These values represent modest departures from Gaussianity, reflecting distributions less right-tailed than those observed in controls. By contrast, non-lesional tissue within the tumoral breast exhibited substantially more pronounced non-Gaussianity. Pooled skewness values were markedly elevated: 4.26 for SUV and 5.06 for *K_i_*, compared to 1.58 for *V_d_*. Kurtosis values were particularly pronounced for SUV and *K_i_* (43.37 and 58.77, respectively), indicating heavy upper tails characteristic of extreme outlier-driven heterogeneity.

**Figure 4:**
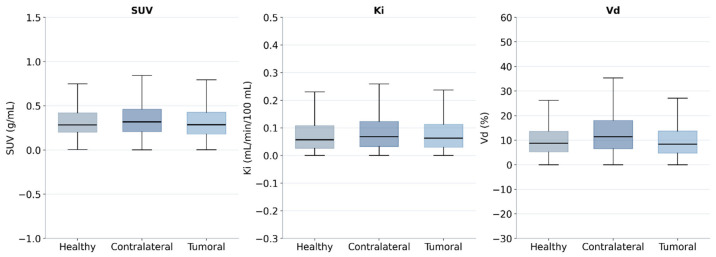
Pooled voxel boxplot of control, contralateral, and tumoral non-lesional breast tissue comparison, in the three metabolic maps: SUV (g/mL), *K_i_* (mL/min/100 mL) and *V_d_* (%).

Despite the noted distributional differences, central tendency estimates revealed substantial overlap between the two non-tumor tissue regions (Figure 4). Population-level pooled median [IQR] values in contralateral breast tissue were: SUV = 0.36 [0.26–0.50] g/mL, *K_i_* = 0.070 [0.034–0.124] mL/min/100 mL, and *V_d_* = 11.98 [6.57–18.57] %. In tumoral non-lesional tissue, pooled median [IQR] values were: SUV = 0.34 [0.24–0.49] g/mL, *K_i_* = 0.073 [0.033–0.130] mL/min/100 mL, and *V_d_* = 9.18 [5.56–13.88] %. A results summary is reported in Table 3.

Despite marked departures from Gaussianity, both contralateral and tumoral non-lesional breast tissue remained statistically concordant with control whole-breast tissue at the population and patient-median levels. Distributional similarity metrics relative to the control reference population revealed high concordance, particularly for *K_i_*:

▪ Contralateral vs. control breast: Jensen–Shannon distances were 0.106 (SUV), 0.100 (*K_i_*), and 0.153 (*V_d_*), with corresponding overlap coefficients of 0.891, 0.910, and 0.847, respectively.
▪ Tumoral non-lesional vs. control breast: Jensen–Shannon distances were 0.119 (SUV), 0.078 (*K_i_*), and 0.054 (*V_d_*), with corresponding overlap coefficients of 0.892, 0.937, and 0.951, respectively. The notably lower *K_i_* Jensen–Shannon distance and high overlap coefficient (0.937) indicate that *K_i_* distributions in tumoral non-lesional tissue are particularly similar to control tissue despite elevated skewness.

These findings indicate that both tissues exhibit median kinetic parameters within the normal breast tissue range, despite small differences in distributional shape.

### 3.4. Parametric Distribution of Tumoral Lesions by BC Subtype

**Figure 5:**
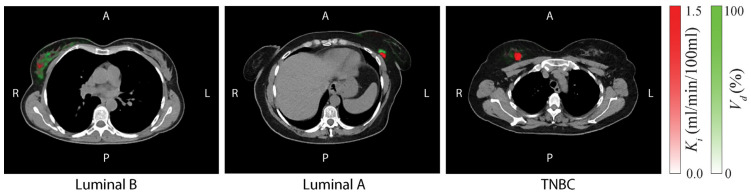
Representative parametric maps of breast lesions across three histological subtypes in this study cohort. Specifically, Luminal-B-like is pt03, Luminal-A-like is pt15, and TNBC is pt06dx. All images are shown using fixed ranges: *K_i_* between 0.0 and 1.5 mL/min/100 mL, and *V_d_* from 0 to 100%. (A = anterior, P = posterior, R = right, L = Left).

**Figure 6:**
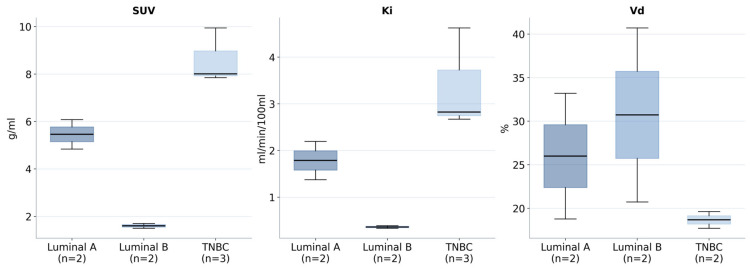
Pooled voxel boxplot of Luminal-A-like, Luminal-B-like, and TNBC lesion, in the three metabolic maps: SUV (g/mL), *K_i_* (mL/min/100 mL) and *V_d_* (%).

Having established the metabolic baseline of control and non-lesional breast tissue in our cohort, we explored lesion-level dynamic kinetic parameters across molecular subtypes (Figure 5). A summary is reported in Table 4 and Figure 6. Given the limited number of patients and lesions, this analysis was intended to describe preliminary patterns. Lesion-level parameters were interpreted primarily in terms of subtype-specific medians, interquartile ranges, and exact nonparametric effect-size-oriented comparisons.

Lesion SUV and *K_i_* both showed a coherent monotonic increase from Luminal B to Luminal A to TNBC. For lesion SUV median, subtype medians were 1.60 g/mL for Luminal B, 5.46 g/mL for Luminal A, and 8.00 g/mL for TNBC. A similar pattern was observed for SUV maximum values (2.70, 9.68, and 13.67 g/mL, respectively) and for intralesional dispersion, as reflected by the standard deviation (0.34, 1.43, and 2.10, respectively). In general, an increasing trend for SUV median, maximum, and standard deviation.

Lesion *K_i_* showed the same ordered behaviors. Median *K_i_* values increased from 0.36 mL/min/100 mL in Luminal B to 1.78 mL/min/100 mL in Luminal A and 2.82 mL/min/100 mL in TNBC. Maximum *K_i_* values similarly increased from 0.96 to 2.98 and 5.41 mL/min/100 mL, while *K_i_* standard deviation increased from 0.15 to 0.59 and 0.97, indicating progressively greater intralesional heterogeneity from Luminal B to Luminal A to TNBC. As for the SUV value, a progression was observed for *K_i_*’s median, maximum, and standard deviation.

When Luminal A and Luminal B lesions were pooled and compared against TNBC, the direction and effect size of the differences remained strong for both SUV and *K_i_*, despite the limited sample size. For the lesion *K_i_* median, the pooled Luminal median was 0.88 mL/min/100 mL versus 2.82 mL/min/100 mL in TNBC, with complete ordinal separation (Cliff’s delta = 1.00; probability of superiority P[TNBC > Luminal] = 1.00). The Hodges–Lehmann location shift for *K_i_* was +2.38 mL/min/100 mL (bootstrap 95% CI, 0.96 to 4.24). For the lesion SUV median, the pooled Luminal median was 3.27 g/mL versus 8.00 g/mL in TNBC, again with complete ordinal separation (Cliff’s delta = 1.00; P[TNBC > Luminal] = 1.00) and a Hodges–Lehmann shift of +5.63 g/mL (bootstrap 95% CI, 2.47 to 7.48).

By contrast, *V_d_* did not show the same monotonic subtype behaviors when summarized only over positive voxels. Positive-only lesion *V_d_* medians were 30.72% in Luminal B, 25.98% in Luminal A, and 19.61% in TNBC, with no ordered trend (exact one-sided *p* = 0.781). Likewise, the occupancy-adjusted *V_d_* burden decreased from 30.09 in Luminal B to 16.60 in Luminal A and 8.16 in TNBC, but without ordered separation (*p* = 0.962). The most informative *V_d_*-derived descriptor was instead the zero fraction, which increased from 0.029 in Luminal B to 0.341 in Luminal A and 0.584 in TNBC.

## 4. Discussion

This proof-of-concept study explored the metabolic patterns of BC across subtypes using dynamic ^18^F-FDG PET/CT with Patlak kinetic analysis. The main observations were: (1) control and non-lesional breast tissue exhibit broadly consistent central metabolic values, with non-Gaussian voxel distributions; (2) lesion-level kinetic parameters showed descriptive differences across the Luminal-B-like → Luminal-A-like → TNBC spectrum; and (3) dynamic kinetic parameters may provide complementary information to SUV value by separating tracer influx from tracer distribution.

The characterization of non-pathological breast tissue is a necessary preliminary step for interpreting dynamic PET-derived parameters. In this cohort, normal breast tissue revealed right-skewed, leptokurtic voxel distributions across all parametric maps—consistent with the known biological heterogeneity of normal breast tissue. Despite this distributional complexity, inter-individual distributions were sufficient to derive preliminary reference distributions for descriptive purposes. The observed median values in control breast tissue were SUV 0.28 g/mL, *K_i_* 0.056 mL/min/100 mL, *V_d_* 9%. Contralateral and non-lesional tumoral breast tissue remained statistically indistinguishable from controls with small differences in distributional shape. These broad overlaps suggest that, at least in this limited dataset, non-lesional tissue generally remained within a similar physiological range.

At the lesion level, the data suggested a possible metabolic gradient across the subtype spectrum. Luminal-B-like lesions in this cohort, characterized by low PgR (<20%) and low proliferation rate (Ki-67 < 20%), exhibited the lowest *K_i_* (median 0.33 mL/min/100 mL) with medium-high *V_d_* (20–40%). Based on the conventional interpretation of Patlak-derived kinetic parameters, we hypothesize that this pattern may reflect relatively low glycolytic metabolic trapping, in the presence of a relatively preserved reversibly exchangeable tracer component, as indicated by medium-to-high *V_d_*. Luminal A-like lesions, characterized by a limited proliferation rate (Ki-67 < 20%) and medium-high PgR (20% and 70%), showed intermediate values for both Patlak-derived parameters (*K_i_* median 1.78 mL/min/100 mL, *V_d_* median ~20–30%). This may suggest an intermediate glucose phosphorylation capacity, compared with non-pathological breast tissue, although this interpretation remains speculative. Finally, TNBC lesions showed the highest *K_i_* (median 3.37 mL/min/100 mL) and a greater proportion of zero-valued *V_d_* voxels. In line with the physiological meaning of *K_i_* as a marker of irreversible FDG trapping, we hypothesize that this pattern may be compatible with more pronounced glycolytic activity. However, the interpretation of *V_d_* is less certain, and zero-valued *V_d_* voxels may also reflect technical factors related to image quality, reconstruction, motion correction, or voxel-wise fitting instability. Despite being completely exploratory, these findings open up hypotheses regarding whether Patlak-derived parameters may capture aspects of tumor biology not fully represented by static uptake measures.

Previous studies provide additional support for the potential relevance of dynamic FDG PET parameters in BC [42,43]. While the field has traditionally focused on whole-body dynamic/parametric PET methodology in mixed-oncology cohorts [44,45], a recent publication by Sundaraiya et al. retrospectively analyzed 24 primary BC lesions in 21 patients using whole-body dynamic FDG PET/CT with Patlak analysis [46]. The authors demonstrated that *K_i_* correlated significantly with histologic grade (grade III vs. II), HER2 status (AUC 0.81), molecular subtypes (AUC 0.81), and hormone receptor status (AUC 0.83), establishing *K_i_* as a promising imaging biomarker of BC aggressiveness.

A second highly relevant study by K. Kajáry et al. examined dynamic FDG-PET/CT in 34 BC patients undergoing initial staging [43]. This group found that *K_i_* was significantly higher in node-positive disease, higher-grade tumors, ER-negative and PR-negative disease, tumors with an elevated Ki-67 proliferation index, and the more aggressive TNBC and HR-negative/HER2-positive subtypes. The authors also reported that stromal tumor-infiltrating lymphocytes were not significantly associated with *K_i_*.

Other less recent work has also highlighted the potential of parametric PET for the early detection of treatment response, in locally advanced breast cancer patients undergoing neoadjuvant chemotherapy [47,48,49]. Collectively, these studies link dynamic FDG kinetic behaviors directly to BC biology and aggressiveness. They support the hypothesis that *K_i_* may serve as a quantitative imaging biomarker of tumor glucose metabolism and proliferative capacity. In contrast, the role of *V_d_* remains poorly characterized in BC. Current literature has not demonstrated consistent associations between *V_d_* and histopathological or clinical features, and its added value beyond *K_i_* alone remains under investigation.

In conclusion, we highlight some important limitations of this study. The BC cohort included only six patients and seven lesions across three molecular subtypes, which limits its statistical power, generalizability, and the ability to account for inter-patient variability. The observed *V_d_* zero-fraction may be influenced by technical limitations, such as reconstruction noise, low-count voxels, motion correction residuals, or voxel-wise fitting instability. Therefore, *V_d_* should be regarded as an exploratory imaging descriptor rather than a validated biological marker. Moreover, the phase of the menstrual cycle may influence the metabolic response, which warrants further investigation in future studies. All findings require independent external validation in larger, prospectively enrolled cohorts with pre-specified statistical plans. Nonetheless, from a clinical perspective, this study demonstrated that the implementation of short-dynamic PET is clinically feasible and could potentially provide clinically meaningful information beyond established imaging, to combine with pathology, and molecular markers.

## 5. Conclusions

This proof-of-concept study provides preliminary estimates of dynamic ^18^F-FDG PET/CT kinetic parameters in normal breast tissue and exploratory difference amongst BC subtype-specific kinetic parameter distributions. Non-pathological breast tissue showed broadly overlapping central SUV, *K_i_*, and *V_d_* values across control and non-lesional regions, whereas tumor lesions showed higher metabolic activity and subtype-associated descriptive patterns. These findings suggest that parametric kinetic maps may help characterize differences between non-lesional and tumor tissue, if validated on larger cohorts.

Future studies should correlate *K_i_* and *V_d_* with quantitative histopathological, molecular, and clinical features to determine whether these imaging parameters capture reproducible aspects of tumor biology. Prospective validation in larger cohorts, using standardized acquisition, reconstruction, segmentation, and statistical analysis protocols, will be necessary before dynamic PET can be considered a clinically applicable, biologically informed imaging strategy in breast cancer.

## Figures and Tables

**Table 1:** Summary of demographic and clinical characteristics in the breast cancer patient cohort.

Patient	Age	Hormone -Receptor	HER2/Neu	Proliferation Activity Ki-67	Grade	Tumor Subtypes *	Menopause
Pt03	50	ER 90%, PgR 10%	score 1+	8%	G2	Luminal B	Pre-
Pt06 dx	59	ER neg, PgR neg	score 0	80%	G2	TNBC	Post-
Pt06 sx		ER 95%, PgR 70%	score 1+	15%		Luminal A	
Pt10	53	ER neg, PgR neg	score 1+	60%	G3	TNBC	Post-
Pt15	64	ER 90%, PgR 20%	score 1+	6%	G2	Luminal A	Post-
Pt20	77	ER neg, PgR neg	score 2+ (FISH-neg)	45%	G3	TNBC	Post-
Pt21	59	ER 95%, PgR 5%	score 2+ (FISH-neg)	12%	G2	Luminal B	Post-

* Subtypes were defined according to St.-Gallen-like immunohistochemical surrogate criteria: Luminal-A-like, ER+/HER2−, PgR ≥ 20%, Ki-67 < 20%; Luminal-B-like (HER2−), ER+/HER2−, PgR < 20% and/or Ki-67 ≥ 20%; TNBC, ER < 1%, PgR < 1%, HER2−. ER, estrogen receptor; PgR, progesterone receptor; HER2, human epidermal growth factor receptor 2; FISH, fluorescence in in situ hybridization; TNBC, triple-negative breast cancer.

**Table 2:** Median values and interquartile range [Q1–Q3] of SUV, *K_i_*, and *V_d_* for control breast tissues. SUV = standardized uptake value; *K_i_* = influx rate constant; *V_d_* = distribution volume.

Control Breast Tissue		
SUV (g/mL)	*K_i_* (mL/min/100 mL)	*V_d_* (%)
0.28 [0.20–0.42]	0.056 [0.026–0.108]	8.72 [5.20–13.62]

**Table 3:** Median values and interquartile range [Q1–Q3] of SUV, *K_i_*, and *V_d_* for non-tumoral breast tissues. SUV = standardized uptake value; *K_i_* = influx rate constant; *V_d_* = distribution volume.

Non-Tumoral Breast Tissue			
	SUV (g/mL)	*K_i_* (mL/min/100 mL)	*V_d_* (%)
Contralateral	0.36 [0.26–0.50]	0.070 [0.034–0.124]	11.98 [6.57–18.57]
Tumoral-breast	0.34 [0.24–0.49]	0.073 [0.033–0.130]	9.18 [5.56–13.88]

**Table 4:** Median values and interquartile range [Q1–Q3] of SUV, *K_i_*, and *V_d_* for tumoral breast tissues. SUV = standardized uptake value; *K_i_* = influx rate constant; *V_d_* = distribution volume.

Tumoral Breast Tissue			
	SUV (g/mL)	*K_i_* (mL/min/100 mL)	*V_d_* (%)
Luminal-B-like	1.68 [1.32–1.79]	0.34 [0.27–0.46]	39 [23–39]
Luminal-A-like	5.72 [4.48–6.73]	1.93 [1.43–2.26]	27 [12–53]
TNBC	8.00 [6.49–9.70]	2.82 [2.29–3.53]	23 [10–34]

## Data Availability

Data associated with this article are stored in the Zenodo repository (zenodo.org) and available upon request due to privacy issues. DOI: 10.5281/zenodo.20719574.
